# Beautiful and Useful: Species Richness and the Ecosystem Services of Allotment Gardens in Berlin, Germany

**DOI:** 10.1002/pei3.70173

**Published:** 2026-06-19

**Authors:** Dagmar Haase, Dara Gaeva

**Affiliations:** ^1^ Department of Geography Humboldt‐University Berlin Berlin Germany; ^2^ Department of Computational Landscape Ecology Helmholtz Centre for Environmental 8 Research – UFZ Leipzig Germany

**Keywords:** Berlin, ecosystem services, melliferous plants, plant species richness, urban allotment gardens

## Abstract

Intensive agriculture and urbanization have led to habitat degradation and fragmentation, reducing plant diversity and subsequently reducing pollinator abundance and richness. This paper investigates the conservation potential of green infrastructure in allotment gardens, which are a common form of urban land use. This exploratory study examines allotment plant species richness by functional use groups in allotment gardens across Berlin, as well as its impact on ecosystem services, such as providing pollinator habitats through multifunctional plants that are edible, ornamental, and nectar‐producing. Additionally, we highlight the potential role of allotment gardens in providing citizens with locally produced food by evaluating the plant species richness of food plants in these gardens. The study was conducted in allotment gardens in Berlin between Мау and July 2019 and focused on comparative analyzes of plots with high (5) and medium (4) management intensity, while the single low‐intensity plot was included only to assess overall biodiversity across all studied garden plots (10). Plant species were identified and classified into functional groups, including edible species, ornamental species, weed species also used as ornamentals, and edible species also used as ornamentals or medicinal plants, as well as into life forms such as herbs, trees, shrubs, and vines. Management intensity was assessed through field observations and by verifying the frequency of lawn mowing and hedge trimming with gardeners. During the study, 376 plant species were identified, 17 of which were included in the list of recommended native plant species for Berlin, while 14 were invasive species. The Wilcoxon rank‐sum test showed that native species richness (*p* = 0.05), weedy plant species richness (*p* = 0.03), and the richness of species contributing to regulation and maintenance ecosystem services (ReMES) (*p* = 0.03) were significantly negatively correlated with the intensity of garden management. Our results show that urban allotments can support pollinator populations thanks to the wide variety of nectar‐producing plants. However, this capacity is greatly affected by how the gardener manages their plot locally. Over half of the identified plants are multifunctional: they are ornamental, providing aesthetic and mental well‐being benefits; they are useful, providing food and medicinal purposes; and they are melliferous, providing nectar and pollen for bees and other insects. Our results clearly show that urban allotments can support pollinator populations due to the high richness of melliferous plants, thereby enhancing the ecosystem services produced in and by gardens that contribute to the physical and mental wellbeing of both gardeners and visitors. We also discuss how this capacity is strongly influenced by local plot management and the gardener's knowledge.

## 1 Introduction

Cities play a vital role in conserving global biodiversity, primarily by designing, managing and actively using urban green spaces (UGSs), which constitute the green infrastructure of urban areas (Pauleit et al. 2019). However, vegetation in today's cities is sometimes poorly structured and unevenly distributed (Andersson et al. 2021). UGS can provide a range of recreational ecosystem services, such as enabling direct contact with nature. However, they are often unavailable to the most vulnerable groups, including children and elderly residents (Sikorska et al. 2020; Haase et al. 2014). For example, children from families with a medium income have fewer green routes to school than children from better‐off families (Łaszkiewicz and Sikorska 2020). Gardens currently represent the main source of biodiversity with which children interact in their daily lives, playing an important role in their connection to nature (Hand et al. 2017).

While gardens are primarily established for recreational purposes and to provide food for urban dwellers, recent studies confirm their role in increasing and maintaining urban ecosystem biodiversity. In Central Europe, for example, allotment gardens have been shown to have high plant species richness and diversity, low synanthropization and valuable geobotanical elements, including species of key importance to the European community (Borysiak et al. 2017). For instance, an agro‐biodiversity survey carried out by the Bundesverband Deutscher Gartenfreunde e.V. in 2008 found and listed 2094 cultivated plant species spreading over 170 plant families in Berlin's allotment gardens (Wagner 2008). Cabral et al. (2017) recorded 290 edible and non‐edible species (excluding those from ornamental areas), including 140 spontaneous species (48%), through field surveys of 0.75 ha across 30 plots in six allotment estates. This ratio of spontaneous species is slightly lower than in recent studies in Manchester (UK) and Poznań (Poland) (Speak et al. 2015), but comparable. Speak et al. (2015) recorded 87 vascular species in Manchester and 375 in Poznań. The list from Poznań included 256 spontaneophytes (72%) and 101 geographic alien species (28%). These proportions signify a very high level of naturalness in the AG flora. In Manchester, UK, more than half of the plants identified were native. However, garden management intensity decreases plant diversity (Delahay et al. 2023). Study by Seitz et al. (2022) on plant diversity in urban community gardens distributed across Berlin grounded documented 404 taxa representing 255 genera.

Urban vegetation in gardens provides a wide range of ecosystem services, primarily food provision, but also cultural and regulatory services (Haase et al. 2014). This paper is one of a small number dedicated to plant biodiversity in European allotment gardens (Speak et al. 2015; Cabral et al. 2017; Borysiak et al. 2017; Klepacki and Kujawska 2018). The study highlights the significance of garden flora biodiversity for the urban ecosystem and the well‐being of its inhabitants, providing an argument for maintaining allotments alongside other modern forms of gardening.

Plant diversity in gardens plays an important role in providing local food for insects, including pollinators. Ahrné et al. (2009) suggest that allotments may serve as an important alternative to missing or declining natural habitats for many bumblebee species, thereby influencing species richness and abundance in the surrounding urban landscape. Overall, urban gardens contribute significantly to the provision of functionally diverse insect communities, including bees, dragonflies, and butterflies, and potentially to urban pollination services (Normandin et al. 2017). A review by Baldock (2020) shows that maintaining suitable habitat areas for pollinators, such as those found in urban allotments, community gardens, and domestic gardens, is key to improving both green spaces and species richness in highly urbanized areas, as it increases floral resources and nesting sites, as well as providing refuges. Gunnarsson and Federsel (2014) conclude that urban gardens contribute to maintaining a high bumblebee population in the centre of cities, indirectly facilitating small‐scale urban food production. The management of gardens to be pollinator‐friendly, with abundant flowering, can support high bumblebee community abundance and diversity. Bee communities and associated pollination services can be maintained and supported in dense urban neighborhoods comprising single‐family and multi‐family homes and gardens, provided these neighborhoods offer diverse and abundant floral resources (Lowenstein et al. 2014).

Baldock et al. (2019) found more plant species in allotments and domestic gardens compared to other urban land uses. This suggests that both native and non‐native plants are important for pollinators foraging in urban areas. However, Fukase and Simons (2016) emphasize the significant impact of the proportion of land dedicated to native species on the number of pollinator visits. Pardee and Philpott (2014) argue that planting flowers native to the region near vegetable gardens could increase bee abundance and potentially enhance the diversity of cavity‐nesting bees.

Using urban gardens as part of a social‐ecological movement can help build a local response to major collapses in urban food supplies (Barthel et al. 2015). Allotments seem especially important for gardeners from disadvantaged backgrounds, as their diets and social networks rely more on and benefit more from their allotments (Veen and Eiter 2018). In terms of the social dimension, the findings of Nordh et al. (2016) and Ferrari et al. (2023) indicate that people participate in urban gardening mainly to create a safe play environment for children, a quiet recreational space for adults, a place to grow things and to have direct contact with nature rather than for biodiversity conservation.

Urban gardens can foster a connection with nature, or ‘biophilia’, in their participants by increasing their exposure to, positive interactions with, and knowledge of nature. This can potentially change people's attitudes towards nature (Lin et al. 2018). Working in gardens with high biodiversity has also been shown to improve the quality of life of young people with autism due to its tremendous therapeutic benefits (Scartazza et al. 2020). Young et al. (2020) report that restoration was positively related to the number of plant species through the perceived restorativeness of the garden. The structural equation model (SEM) suggested that the number of plant species displayed a positive direct association with perceived restorativeness (β = 0.184, *p* = 0.003). Additionally, growing garden plants, including food and medicinal plants, is appealing to children and serves an important educational purpose. Research shows that urban dwellers, including young people, have a need for gardening (Lee et al. 2018). Recent studies of urban gardens have explored the social importance of such areas as places of recreation and food sources, as well as their role in providing refuge for numerous plant and insect species.

This paper aims to investigate plant diversity in Berlin allotment gardens and the impact of garden management on this diversity. Additionally, we aim to demonstrate the link between plant biodiversity and key urban ecosystem services (Haase et al. 2014; Haase and Wolff 2022). Particular focus is given to melliferous allotment garden plants, which are a vital food source for pollinating insects in urban ecosystems.

Based on this knowledge, our study hypothesizes that (i) the intensity of garden management affects plant species richness. Studies examining the biodiversity of pollinator insects and the flora in urban green spaces support the hypothesis (ii) that allotments can support a variety of melliferous plants, with many garden plant species being crucial for pollinators. (iii) In addition, we can hypothesize that most plant species grown in allotments are multifunctional and can provide multiple ecosystem services simultaneously.

## 2 Material and Methods

### 2.1 Study Area and Data Collection

**FIGURE 1:**
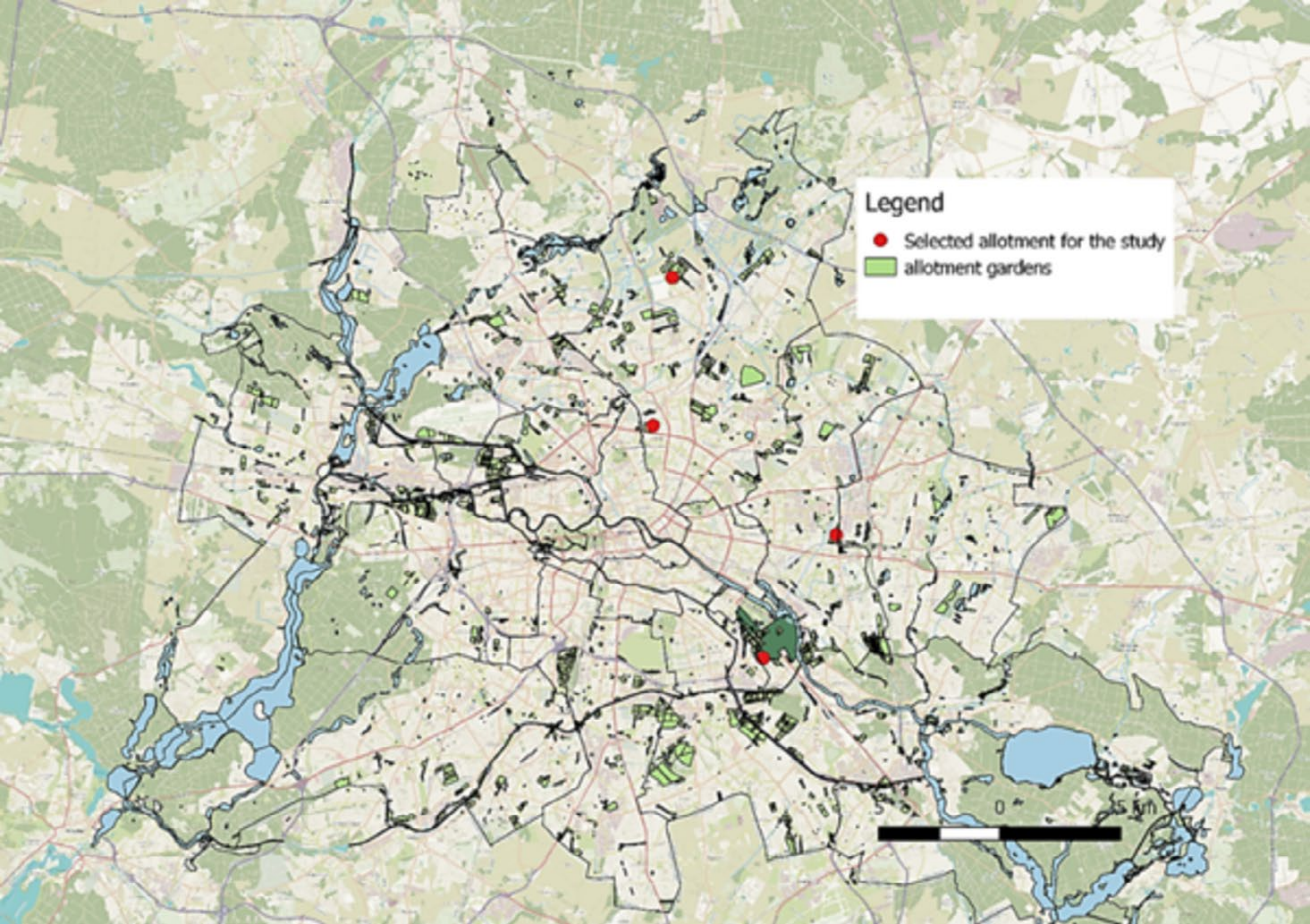
Location of the studied allotment gardens in Berlin (own design using Esri Deutschland (2020) and OpenStreetMap).

For the survey, which was carried out between May and July 2019, 10 garden plots were selected from four allotment associations, covering a total area of around 0.4 ha in Berlin, Germany (see Figure 1). During the field study, data were collected on the number of plant species in each plot. We identified all plant species on studied allotment plots which were further classified in five functional groups: (1) edible, (2) ornamentals, (3) weeds, (4) weed species also used as ornamental, and (5) edible species used also as ornamentals or/and as medicinal.

For the inventory of plant species richness in plots with dense herbaceous cover (lawns and weed cover), plant species were recorded through continuous surveying along a diagonal transect line. During the survey, all encountered plant species were recorded without the use of sampling quadrats. Species were visually recorded within a narrow belt approximately 20 cm on either side along the transect line. For each species encountered, its presence along the transect was documented.

For trees, shrubs, plants on ornamental flowerbeds, and vegetable beds, a complete species inventory was conducted. Due to the need to preserve the cultivated plantings and the impossibility of direct access through them, surveys of flower and vegetable beds were conducted using a visual inspection from the periphery. One limitation of this approach is that some species may have been overlooked during the survey process. Each plot was surveyed twice, during spring month (May) and in summer (Juny‐July).

The allocation of plants into groups was based on reference data from literature: Pflanzen für Berlin: Verwendung gebietseigener Herkünfte (2013); Bienenfreundliche Pflanzen für Balkon und Garten (2017); Wildblumen zur Förderung von Wildbienen (2019); FloraWeb (2020); Bienenfreundlicher Garten (2021); Denisow and Wrzesien (2007); Mačukanović‐Jocić and Jarić (2016); Bufe and Korevaar (2018); Ion et al. (2018); Jachuła et al. (2019); Karamaouna et al. (2019); and finally, Rollings and Goulson (2019). We also identified melliferous and native species among all species. In this context, ‘native’ species refers to plants that originate from populations of native species which have reproduced within a natural area over multiple generations and a long period of time. Genetic differentiation from populations of the same species in other natural areas can be assumed (Pflanzen für Berlin: Verwendung gebietseigener Herkünfte 2013, 40, according to BMU, 2012). Collection, preparation of data, and all further data analyzes and graphical presentations were performed using Microsoft Excel and R (version 4.3.0).

Our research is based on recent studies of allotment and community gardens conducted in Poland, the UK, Germany, and the US. Table 1 summarizes the most relevant studies on plant richness and ecosystem services in urban gardens, which are most relevant to the approach used in the present study. In these studies, the authors used both remote and field methods to assess garden biodiversity, focusing on gardens in one or two cities. Regarding the study of plant species richness in relation to the provision of ecosystem services, the study by Speak et al. (2015) is notable, as it included a detailed land cover characterization combined with an assessment of the floral diversity of allotment gardens. Cabral et al. (2017) evaluated plant species richness in allotment and community gardens, categorizing them according to the intensity of garden management. Additionally, the study by Clarke and Jenerette (2015) investigated biodiversity in relation to useful plant groups and their contribution to ecosystem services. Our study primarily investigated plant species richness in garden plots and established differences in plant species richness by use group across high and medium garden management intensity. Our analysis primarily addresses the lack of emphasis on the richness of meliferous plants, including ornamental ones.

**TABLE 1:** Sample studies of allotment garden plant biodiversity and ecosystem services related to plant biodiversity in urban allotments and community gardens.

References	Ecosystem service	Indicator	Overall indicated plant species	Type of garden (AG*, CG**)	City (country)	Number of AG plots*/CG**
Speak et al. (2015)	Provisioning services Regulating services	Variety of vegetables and fruits grown on the plot floral diversity	87	AG	Manchester (UK)	497*
			357	AG	Poznan (PL)	1164*
Clarke and Jenerette (2015)	Provisioning services Cultural services	Plant biodiversity according to major use categories (ornamental, medicinal, edible)	707	CG	Los Angeles (USA)	14**
Cabral et al. (2017)	Provisioning services	Number of edible species grown on the plot; difference in species composition	290	AG/CG	Leipzig (DE)	30*/6**

* AG, allotment garden.

** CG, community garden.

In our study, we randomly selected the plots, as suggested in the study by Borysiak et al. (2017), provided that the gardeners agreed. The plot selection followed a two‐step approach based on formal access. First, initial contact was established via written correspondence with the chairpersons and individual owners of allotment gardens across Berlin. The sampling pool was restricted to plots where explicit written permission for field surveys was granted. Then potentially suitable gardens during exploratory field visits were identified. We initially contacted the garden owners by email to arrange a visit.

Second, a preliminary visual inspection of the accessible allotments was conducted to evaluate baseline management features (e.g., status of lawns, hedges, and beds). Based on this inspection, the plots were categorized into three distinct groups of management intensity. Finally, within each pre‐defined group, individual plots were randomly selected for comprehensive floristic inventory. All of the garden associations that we studied were located within the city limits, within 100 m of the edge of the built‐up area. Following Cabral et al. (2017), we identified three plot types ranging from high to medium to low intensity. Although we did not conduct a questionnaire survey, we asked gardeners how often they mowed their lawns and trimmed their hedges to visually identify a group of plots. Nine plots were selected for comparative biodiversity analysis in four allotment estates, consisting of five high‐intensity plots and four medium‐intensity plots. In the high‐intensity plots, the grass was cut almost weekly and the hedges were cut once a year. In the medium‐intensity plots, the hedges were usually not cut radically, but at least once a year, and the lawns were mown every other week. In the low‐intensity plots, hedges were cut once a year (or individual sections of hedges every two years) and lawns were mown less than once a month.

A **high‐intensity** plot was defined as one with a high apparent level of maintenance. This included weeding, mowing, and pruning of all available land, and the rare occurrence of spontaneous vegetation and weeds across the entire plot. There was also an absence of flowers on the hedge, indicating frequent cutting when using flowering shrubs. A **medium‐intensity** plot was characterized by infrequent lawn mowing and hedge trimming, with spontaneous vegetation and weeds present on all parts of the plot, and rare flowers on the hedge and lawn. A **low‐intensity plot** was defined as one where the lawn is rarely or never mowed, with spontaneous plants dominating and vegetation blooming and forming seeds on the lawn and hedges. Only one low‐intensity plot was available for the survey. Therefore, it was included in the study solely to assess the overall biodiversity of the gardens, but it was not used in the statistical comparison with medium‐ and high‐intensity management plots. Data from the low‐intensity plot were retained to estimate the total species richness across the 10 studied garden plots of Berlin allotments.

The data values are independent. The plant richness in medium‐intensity gardens does not depend on the plant richness in high‐intensity gardens. We assume that the surviving garden plots represent a simple random sample of all allotment plots in Berlin. Firstly, we conducted descriptive analyzes to summarize the relationship between the number of plant species and the management intensity of the allotment plots. We then compared the species richness of medium‐ and high‐intensity management plots (*n* = 9) within the groups of weedy and native plants, plant species by type of use, and plant groups contributing to different ecosystem services.

### 2.2 Plant Classification

- No standardized questionnaire survey or guided walks with gardeners were conducted. The functional classification of plant species e.g., ornamental, edible, weeds with ornamental value, pollinator‐friendly plants (meliferous) was performed by the authors during field surveys, based on the observed use of plants within the plots and on the authors' horticultural and ecological expertise.

All plant species were identified directly in the gardens or after survey (with use of photos) by our own after obtaining permission to access the plots. The classification on the functionality and use of plant species was based on our own expertise and did not rely on gardeners' reported information.

**FIGURE 2:**
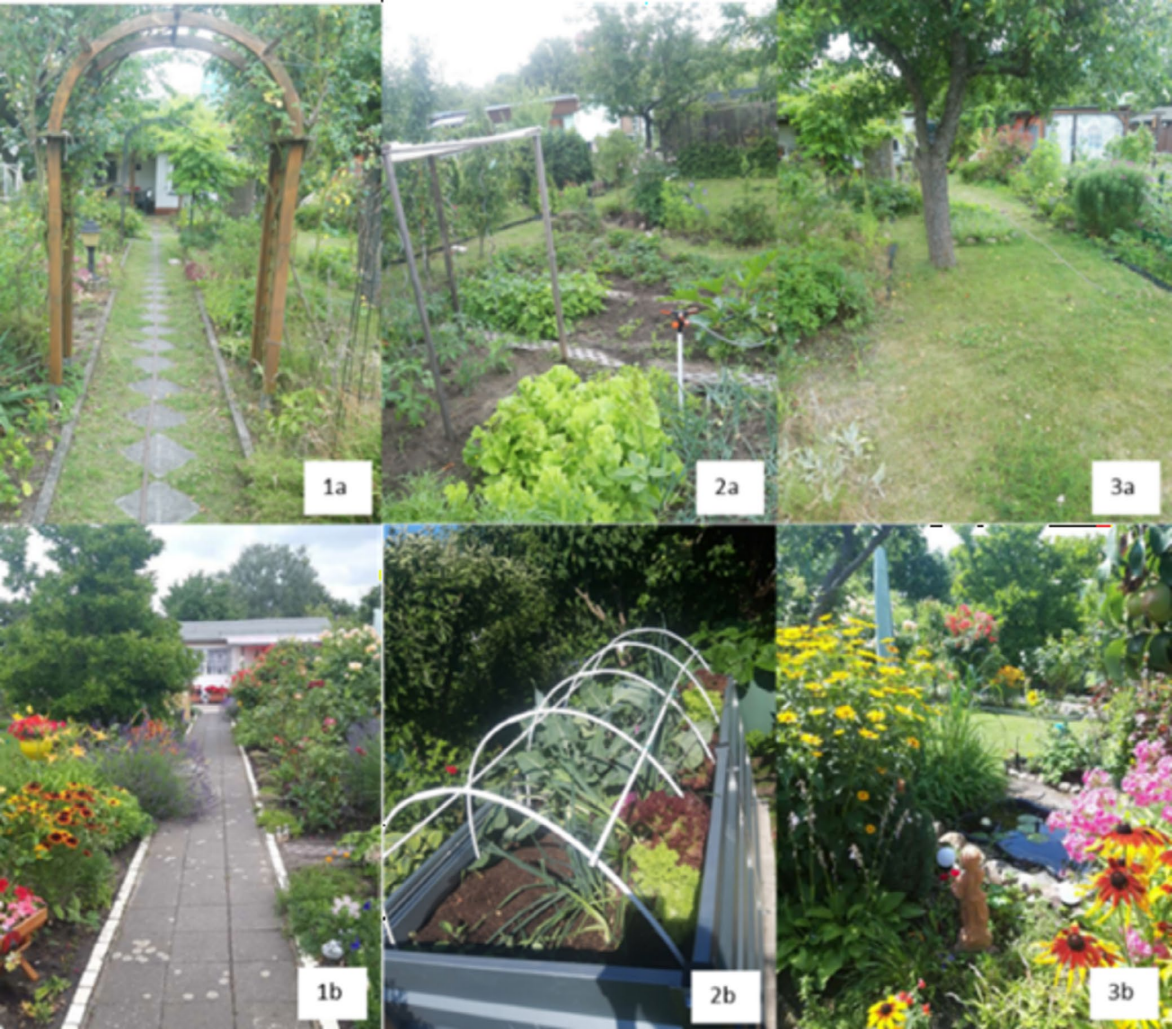
Types of plots included in comparative analysis: 1a‐3a—medium intensity plot; 1b‐3b—high‐intensity plot; (from left to right: Path area; edible crops; ornamental plants and lawn) (photos by the authors).

In 10 plots across four sampled allotment estates, we identified all plants according to their life form (trees, shrubs, vines, or herbaceous plants), based on multiple classifications of urban plants by Avolio et al. (Avolio et al. 2020; Figure 2; Table S2). We then categorized the plants as either spontaneous/weed (not planted) or cultivated (intentionally planted). Additionally, we divided the plant species into broad use categories based on the provision of ecosystem services, including provisioning, regulating and maintaining, and cultural services (Clarke and Jenerette 2015). This included categorizing edible, melliferous, medicinal, and ornamental plants.

In our study, we used the Common International Classification of Ecosystem Services (CICES 2018) to categorize plants according to the type of ecosystem service they provide. We distinguished between the following categories:

- Provisioning: all the nutritive and non‐nutritive material and energy outputs of living systems, as well as abiotic outputs (including water).
- Regulatory and maintenance: all the ways in which living organisms can mediate or moderate the environment, affecting human health, safety, or comfort, as well as abiotic equivalents.
- Cultural: all the non‐material outputs of ecosystems (biotic and abiotic), which are usually non‐rival and non‐consumptive, affecting the physical and mental states of humans.

We included all food plants in the ‘Provisioning’ group, all melliferous plants in the ‘Regulation and Maintenance’ group, and all ornamental and edible plants used in traditional medicine in the ‘Cultural’ group. We also determined whether each plant species was native to Berlin (FloraWeb 2020; ‘Pflanzen für Berlin: Verwendung gebietseigener Herkünfte’ 2013). Some of the plants were identified later from photographs taken during the field survey of the plot in cases where identification on site appeared difficult (see again Figure 2).

### 2.3 Statistical Analysis

We used a Wilcoxon rank‐sum test to investigate differences in plant species richness variables between allotment gardens with high and medium management intensity. This statistical test was chosen because the sample sizes are small (*n* = 5 for high management intensity and *n* = 4 for medium management intensity), and it is a more appropriate method for small sample sizes where normal distribution is not assumed. We compared plant richness in total, across native plants, weeds, and melliferous species, invasive species, as well as in groups according to the ecosystem services they provide. To measure the effect size (r), showing how large the difference is between the distributions of the high‐ and medium‐intensity plot groups of gardens, the rank biserial correlation coefficient (Swacha et al. 2024) was computed. The effect size was calculated using the formula

$$r=Z/\sqrt{N},$$

where *Z* is the standardized test statistic (*z*‐score) and *N* the sample size (Fritz et al. 2012). According to (Coolican 2009, 395 cited in Fritz et al. 2012), a large effect (*r* > 0.5). Large effect sizes were observed for native species richness, weedy plant richness, and species contributing to ReMES. The following species groups were observed: native species, weeds, and plant species contributing to ReMES and ProvES+ReMES+CES.

When conducting Wilcoxon rank sum tests to compare plant species richness between garden management types and various functional and ecosystem service‐based groups of plant species, we relied on our previously proposed hypotheses and made no more than 14 comparisons. To avoid Type II errors, we did not apply any adjustment for multiple comparisons following Perneger (1998). The test was conducted based on a complete list of species for each plot, which was subsequently divided into species groups.

To identify the plant species that are characteristic of plots with different degrees of management, we used the IndVal package in R to calculate the IndVal index (indicspecies package) (Bellini et al. 2025). We also checked the statistical significance (p‐value) of species with an IndVal close to one using the permutation method with the indicspecies:multipatt function in R.

## 3 Results

### 3.1 Total Plant Species Richness

**FIGURE 3:**
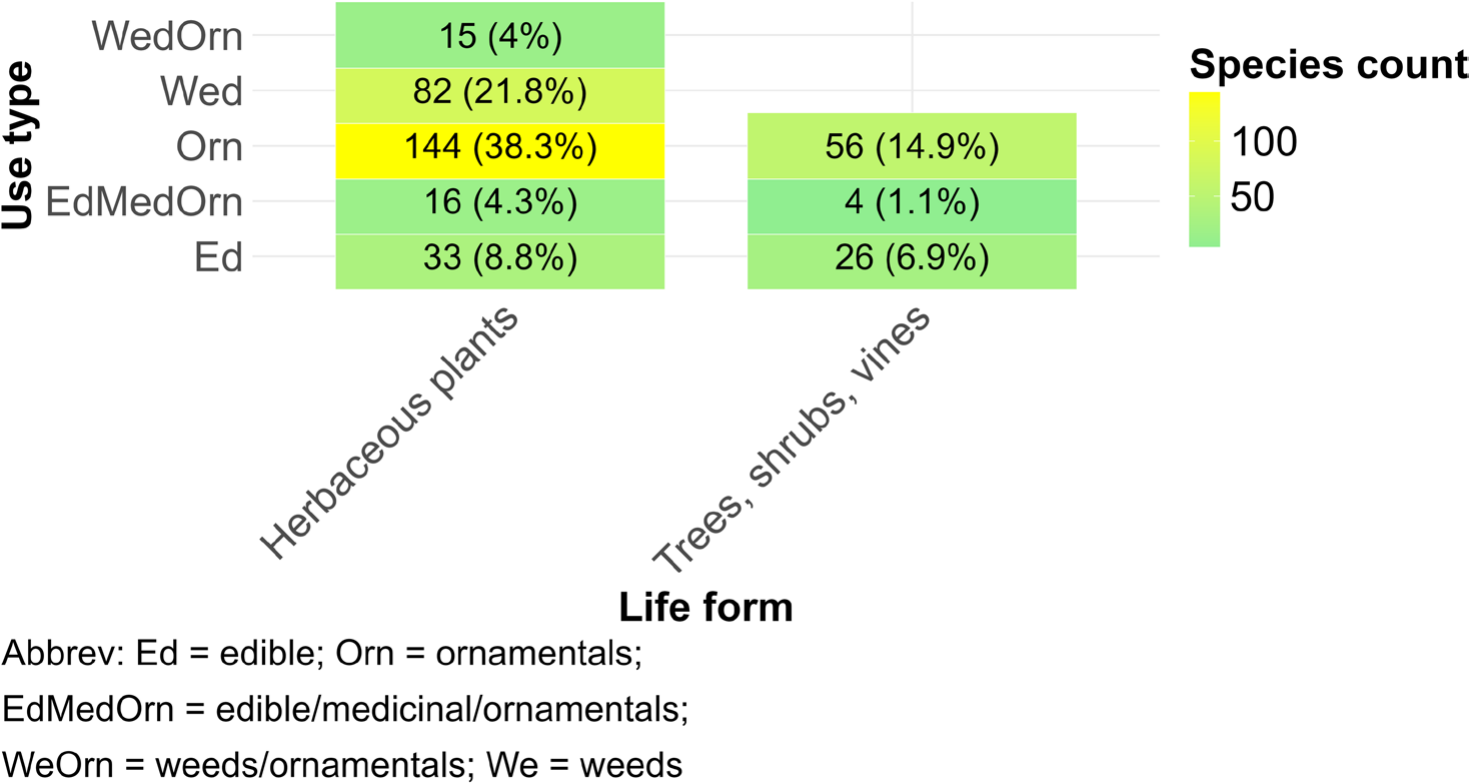
The number and proportion of all identified plant species for the main use categories and Weeds (spontaneous plants).

In this pilot study, we identified 377 plant species (see Table S1), 17 of which were included in the list of recommended native plant species for Berlin (Pflanzen für Berlin: Verwendung gebietseigener Herkünfte 2013). Where there were no flowers or the plant had already been cut, species were identified up to genus level. The plot sizes in the allotments studied ranged from 430 to 277 m^2^ (see Table S1). Most of the species were ornamental plants (201), followed by weeds (82) and edible plants (59). Twenty species can be used in folk medicine as well as for ornamental and edible purposes. The remaining species (15) belong to the ‘weedy and ornamental’ group—species that can be found as both weeds and ornamentals on plots. Ornamentals are primarily plants grown for their aesthetic beauty. Our research revealed that 54% of plant species are used as ornamentals. Additionally, we identified eight edible plant species that can be used in folk medicine, and four that are used as both ornamentals and medicines (Figure 3).

### 3.2 Distribution of Species by Family and Use Type

Of all the species identified, the largest number of edible herbs belonged to the Lamiaceae family, while the largest number of edible woody plants belonged to the Rosaceae family. Most ornamental grass and weed species belonged to the Asteraceae family (see Table S3).

All identified plants belonged to 81 plant families (Table S3). The most abundant families in terms of species number were *Asteraceae* with 50 species, *Rosaceae* with 38 species, *Lamiaceae* with 24 species, and *Poaceae* with 12 species. Thirty‐four (40%) of the plant families were made up of only one species. The 15 families with more than five species were the most abundant in terms of species number (Figure 3). The Asteraceae family had the most ornamental species (26), followed by the Rosaceae family with the most weed species (17), and the *Lamiaceae* family with the most edible species (16). The group of species that are both edible and used in folk medicine and as ornamentals included eight *Lamiaceae* species. Many melliferous species belonged to the *Asteraceae, Rosaceae*, and *Lamiaceae* families.

The most common species, which occurred in six to ten of the ten plots sampled, are listed in Table S6. Herbaceous edibles in most gardens were mainly represented by *Phaseolus vulgaris L*. (Fabaceae), *Melissa officinalis L*. (Lamiaceae), *Cucumis sativus L*. (Cucurbitaceae), *Cucurbita pepo L*. (Cucurbitaceae), *Solanum lycopersicum L*. (Solanaceae), *Brassica oleracea L*. (Brassicaceae), *Solanum tuberosum L*. (Solanaceae), *Lactuca sativa L*. (Asteraceae), *Rheum L*. (Polygonaceae), *Fragaria ananassa (Duchesne ex Weston) Rozier* (Rosaceae), *Satureja hortensis L*. (Lamiaceae), *Daucus carota subsp. sativus* (Apiaceae) and *Petroselinum crispum (Mill.) A. W. Hill* (Apiaceae), as well as *Allium cepa L*. (Amaryllidaceae) and *Allium schoenoprasum L*. (Amaryllidaceae).

The most abundant family of woody fruit plants, Rosaceae, was represented in most gardens by species such as *Malus domestica (Suckow) Borkh*., *Prunus domestica L*., *Rubus fruticosus L., s. l*., *Prunus cerasus L*., *Ribes rubrum L*. and *Prunus avium (L.) L*. Pyrus communis was also common and was found in most of the surveyed plots. On average, 13 herbaceous and 10 woody (trees, shrubs and vines) edible plant species were cultivated per plot. The most common ornamental species were the herbaceous plants *Paeonia officinalis L*., *Rosa L*., *Hydrangea macrophylla (Thunb. ex Murray) Ser*. and *Ligustrum vulgare L*., and the woody plants *Hedera helix L*., *Syringa vulgaris L*., *Iris L*. and *Lavandula angustifolia Mill*., *Hydrangea macrophylla (Thunb. ex Murray) Ser*. and *Ligustrum vulgare* L. The most common weed species were *Lolium perenne L*., *Taraxacum sect. Ruderalia Kirschner, H. Øllg. & Štěpánek*, *Aegopodium podagraria L., Trifolium repens L., Stellaria media (L.) Vill*. and *Veronica filiformis Sm*. The most common species in the group of multifunctional species that are simultaneously edible, ornamental and/or medicinal was *Melissa officinalis L*. Among the weed species found as ornamentals, the most common plant species was *Bellis perennis L*. The most common local species were *B. perennis* , *H. helix* and *R. rubrum* .

**FIGURE 4:**
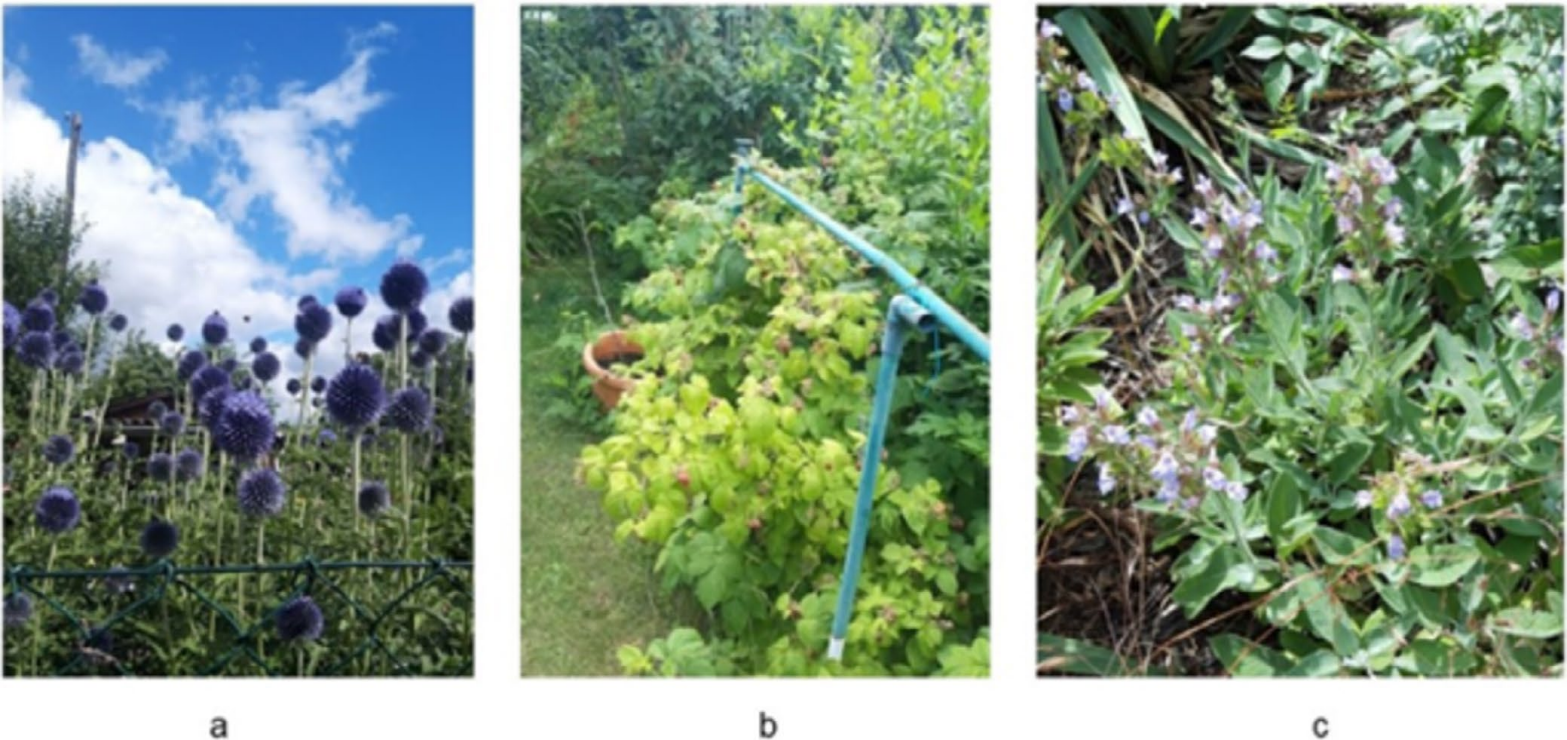
Representatives of the garden multifunctional plant species (а) *Echinops ritro L*. (Asteraceae; ornamental, habitat provision); (b) *Rubus idaeus L*. (Rosaceae; food, habitat provision); (c) *Salvia officinalis L*. (Lamiaceae; food (spicy herbs), folk medicine, habitat provision) (own pictures, Berlin, 2019).

Moreover, many ornamental, edible, and folk medicinal plants are often beneficial to insects (Figure 4). For example, it was found that the total amount of sugars secreted by 10 flowers was approximately 40 mg for *Echinops ritro L*. and *Rubus idaeus L*. (Kostryco and Chwil 2022; Jabłoński and Kołtowski 2005), and 4–20 mg for species in the Salvia genus (Jabłoński and Kołtowski 2005). The most common melliferous species were *Paeonia officinalis L*., *Rosa L*., *Ligustrum vulgare L*., *Aquilegia vulgaris L*., *Melissa officinalis L*., *Syringa vulgaris L*., *Iris L*., *Lavandula angustifolia Mill*., *Alcea rosea L*., *Calendula officinalis L*., *Centaurea dealbata Willd*., *Hemerocallis fulva L*., *Hibiscus syriacus L*., and *Rhododendron L*. At the same time, only non‐double flower forms of ornamental species were considered beneficial to insects.

**FIGURE 5:**
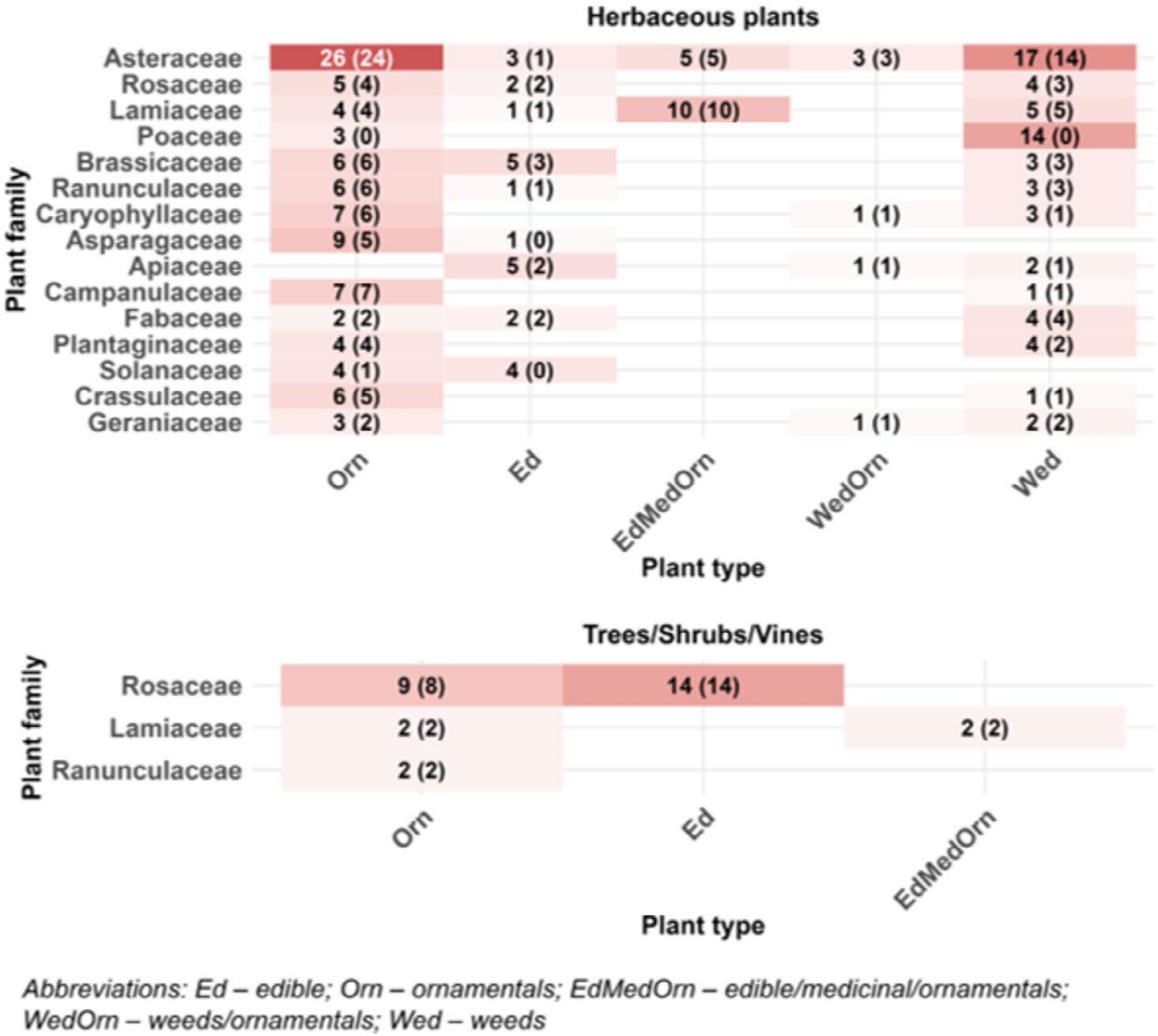
The top 15 plant families with more than five species, divided by the herbaceous, tree, shrub, and vine groups, and the type of plant use, with the percentage of melliferous and polliniferous plant species shown in brackets.

The number of melliferous species was similar for weeds and food plants, at 51 and 55 respectively. Furthermore, the number of melliferous species was similar for both ornamental and food plants, as well as for ornamental and weed plants. Melliferous (polleniferous) plants are a source of nectar and/or pollen for insect pollinators, mainly belonging to the Asteraceae, Rosaceae, Lamiaceae, Fabaceae, and Boraginaceae families. However, some melliferous plants are not attractive to honeybees (*Apis mellifera L*.) due to the peculiarities of their flower structure. Nevertheless, they may be a potential source of nectar or pollen for wild bees, for example—bumblebees (*Bombus*) (Verweij et al. 2025). More than half of the ornamental plants, as well as most of the cultivated and weed plants, identified in our study were melliferous (polleniferous). The total number of native plant species in the entire study area was 37 (9% of the total), 29 of which (78.4%) were melliferous (polleniferous; Figure 5).

### 3.3 Ecosystem Services

**FIGURE 6:**
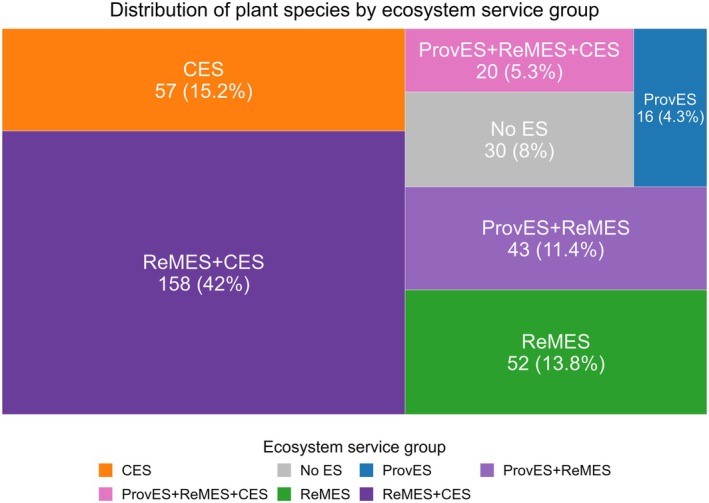
Plant species richness based on the ecosystem service classification (CES—cultural ecocystem servieces; ProvES—provisional ecocystem servieces; ReMES regulational and maintenance ecocystem servieces; ProvES + ReMes provisional and regulational and maintenance ecocystem servieces; ReMES + CES regulational and maintenance and cultural ecocystem servieces; ProvES + ReMES + CES—provisional and regulational and maintenance and cultural ecocystem services; No ES = species with no assigned ecosystem service).

Based on the identified plant groups, we categorized all the identified plant species according to their contribution to different types of ecosystem services. These groups are: (a) cultural: ornamental and edible plants used in folk medicine, (b) provisional: edible plant species and (c) regulation and maintenance: melliferous weedy plants. Additionally, we identified three groups of plants that contribute to multiple types of services: provisional/regulation and maintenance—edible melliferous plants; provisional/regulation and maintenance/cultural—edible, ornamental (or used in folk medicine) melliferous plants; and the largest group, regulation and maintenance/cultural, which includes ornamental or folk medicine melliferous plants (Figure 6).

Plants related to CES that were used solely for decorative purposes or in folk medicine accounted for 15% of the total. The largest share (42%) was occupied by plants related to the regulation and maintenance of cultural ecosystem services (ReMES+CES). About 14% were predominantly weed melliferous plants related to the regulation and maintenance of ecosystem services (ReMES). 11% were edible melliferous plants related to provisional, regulatory, and maintenance ecosystem services (ProvES+ReMES). 20 species (5%) were plants used for food and in folk medicine and/or as ornaments, which are related to provisional, regulatory, maintenance, and cultural ecosystem services (ProvES+ReMES+CES). Plants grown only for food, not melliferous or ornamental purposes, accounted for 16 species, or 4%. Meanwhile, weed plants (30 species), which do not contribute to the types of service under consideration, represented only 8% of all the plants identified. Of the allotment plants sampled, 271 (approximately 70%) were melliferous. Thus, most of the identified plants (58%) contribute to more than one type of ecosystem service.

### 3.4 Plant Richness and Garden Management Intensity

#### 3.4.1 Species Richness

**FIGURE 7:**
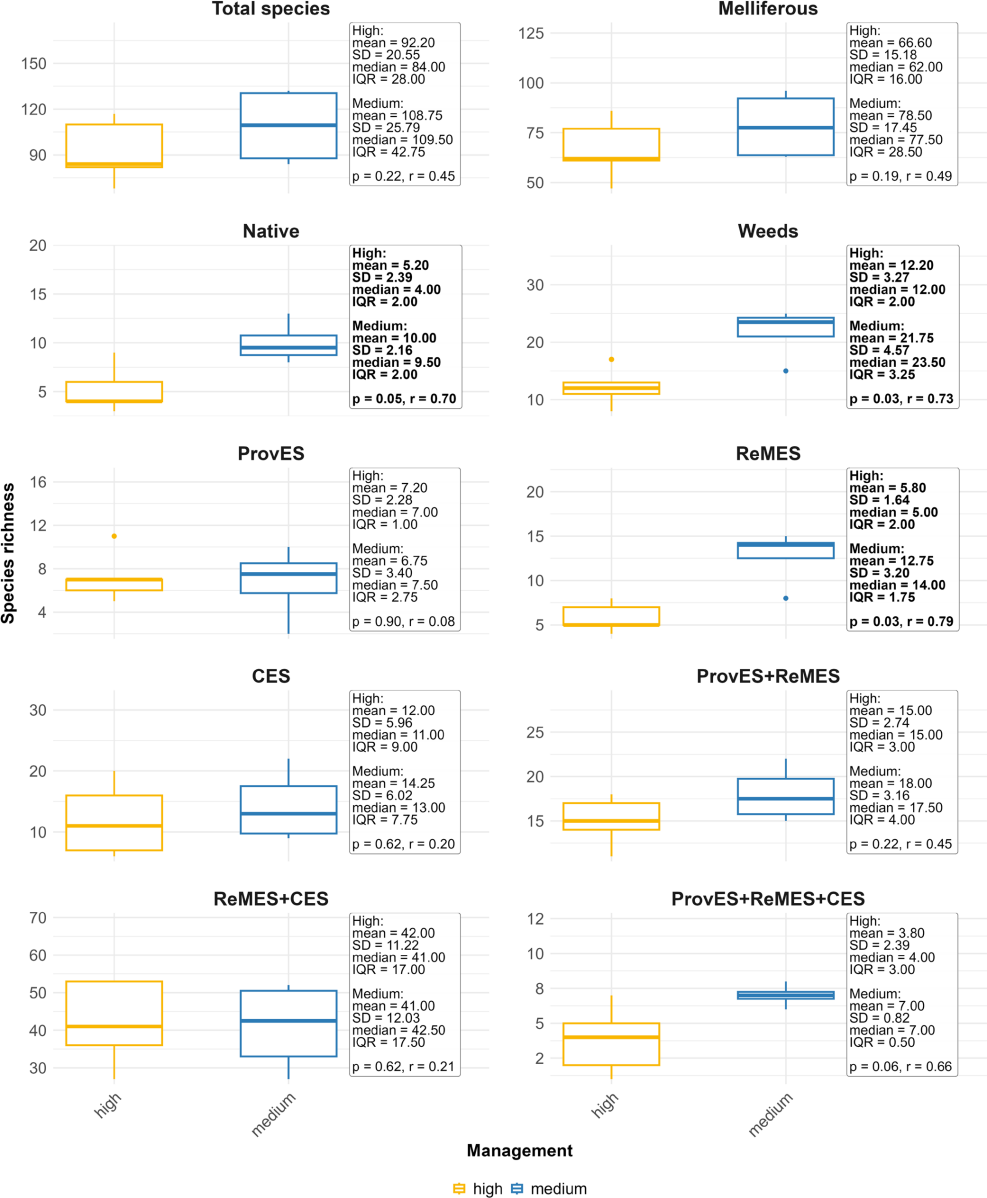
Boxplots illustrating plant species richness scores (species number) in allotment plots with high and medium management intensity: Total species number; native plants, melliferous, plants that contribute to cultural ecosystem services (CES); provisional ecosystem services (ProvES); regulation and maintenance ecosystem services (ReMES); provisional and regulation and maintenance ecosystem services (ProvES + ReMES); regulation and maintenance and cultural ecosystem services (ReMES + CES); provisional and regulation and maintenance and cultural ecosystem services (ProvES + ReMES + CES).

Figure 7 shows a comparison of plant species richness (i.e., the number of species) in plots with high and medium management intensity, as well as the total number of species, native species, melliferous species, weed species, and species responsible for different ecosystem services. All 10 pairs of box plots show a trend towards higher median plant richness in plots with medium management intensity. Also, the distribution of plant species numbers was generally more symmetrical in medium‐intensity plots than in high‐intensity plots, except for the weed species group and the species responsible for regulating and maintaining ecosystems. In plots with a high level of management, the most symmetrical distribution was observed for weed species and species responsible for cultural services.

The most significant median difference between plots with medium and high management intensity was observed for melliferous plants (78 and 62), native species (9.5 and 4), and species responsible for regulating and maintaining ecosystems (10 and 5). Few differences were observed in total plant species richness (84 and 110), as well as in species responsible for provisioning, regulating, maintaining, and cultural ecosystem services (4 and 7). The most homogeneous values in the samples for medium‐intensity management plots were for total species number, melliferous plants, and weeds. The largest difference between extreme values and the median for this type of plot was observed for native species and species responsible for provisioning, cultural, regulatory and maintenance, and cultural ecosystem services.

The most symmetrical distribution was found in the group of weed species and species responsible for cultural services in plots with a high level of management, and the smallest deviation of extreme values from the median was found for species responsible for provisioning, regulating, and maintaining ecosystem services. Therefore, the main differences between plots with an average level of management and those with a high level of management are higher overall plant richness and a clear trend towards greater biodiversity among native, melliferous, weed, and species responsible for regulating and maintaining ecosystems. Conversely, the smallest differences were observed in groups of species responsible for cultural, provisioning, and regulatory and maintenance ecosystem services.

In plots with a high level of management, the median for the total number of species, melliferous species, native species, and species contributing to regulation and maintenance ecosystem services (ReMES) is located at the lower boundary of the boxplot. This indicates a compression of the distribution in the area of low plant species richness values. Therefore, the most intensively managed areas are characterized by poorer species composition for the specified groups of species, with high values rarely occurring. In the group of species contributing to provisioning ecosystem services, the median is located at the upper boundary of the IQR, reflecting an accumulation of values in the high range. This may be the result of the preferred cultivation of edible plant species. For plots with medium‐intensity management of the regulation and maintenance ecosystem service plant group, the median shifts to the upper limit of the boxplot and indicates dominance of higher values in the distribution. This pattern may be a consequence of maintaining conditions that preserve species richness among wild weeds useful for pollinators.

The Wilcoxon rank‐sum test revealed significant results for plant species richness in native species and weedy plants (*p* = 0.05 and *p* = 0.03, respectively). Additionally, the intensity of garden management influenced the number of species contributing to regulation and maintenance ecosystem services (ReMES) (*p* = 0.03) (Figure 7).

Large effect size (*r* > 0.5) was observed as the plant species group belonged to provisional and regulation and maintenance and cultural ecosystem services (ProvES+ReMES+CES).

#### 3.4.2 Invasive Species

Among the identified garden plant species, we also detected 14 species that are invasive in the studied region. According to the German Federal Agency for Nature Conservation (BfN) (Naturschutzfachliche Invasivitätsbewertungen 2025) recorded non‐native species belonged to tree invasion‐status categories. In management list were included species such as *Lupinus polyphyllus Lindl*., *Solidago canadensis L*., *Symphyotrichum novi‐belgii (L.) G. L. Nesom*, and *Syringa vulgaris L*., while *Buddleja davidii Franch*., *Cotoneaster dammeri Schneid., Cotoneaster horizontalis Decne., Erigeron annuus (L.) Desf. and Prunus laurocerasus L*. are classified as requiring management attention. Other species: *Mahonia aquifolium (Pursh) Nutt*., *Miscanthus sinensis (Thunb.) Andersson, Oenothera biennis L., s. str., Parthenocissus quinquefolia (L.) Planch*., and *Symphoricarpos albus (L.) S. F. Blake*, are included in observation or monitoring lists as potentially invasive or naturalized neophytes in urban ecosystems. Median number invasive species number was higher in medium‐managed plots (5.0 species per plot) than in high managed plots (2.0 species per plot); however, the difference was not statistically significant (*p* = 0.30). Among the invasive species included in management lists, four species— *Lupinus polyphyllus* , *Solidago canadensis L*., *Symphyotrichum novi‐belgii (L.) G. L. Nesom*, and *Syringa vulgaris L*—were recorded in plots with medium management intensity, whereas only two species, *Solidago canadensis L*. and *Syringa vulgaris L*., were found in high managed plots.

#### 3.4.3 Indicator and Common Species

**FIGURE 8:**
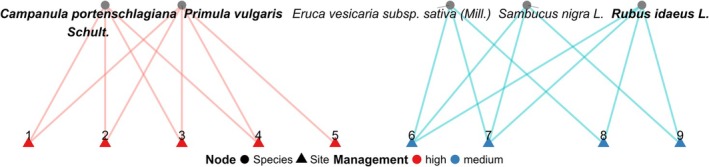
Bipartite network of plant species and surveyed allotment plots: Species with *p*‐value *p* ≤ 0.1 are shown; (plant species names with *p* ≤ 0.05 are in bold); blue and red colors indicate garden management intensity; edges connect plant species to the associated plots.

Of the plant species found in the compared groups of plots, 103 (29%) and 85 (24%) were found only in plots with medium and high management, respectively. A total of 162 species (46%) were shared between the two groups (see Table S4). According to the calculation of the IndVal index three species were found to be indicator species: *Primula vulgaris* *Huds*. for high management and *Eruca vesicaria* *subsp. sativa (Mill.)* and *Rubus idaeus* *L*. for medium management (*p*‐value < 0.05) (Table S5; Figure 8). Fourteen of the most common species were also identified*: Hydrangea macrophylla (Thunb. ex Murray) Ser*., *Aegopodium podagraria L., Aquilegia vulgaris L. s. str., Bellis perennis L., Ligustrum vulgare L., Lolium perenne L., Malus domestica (Suckow) Borkh*., *Prunus avium* *L., Prunus domestica L*., *Solanum lycopersicum L*. and *Taraxacum sect. Ruderalia* and *Trifolium repens* *L*., two of which ( *Paeonia officinalis* *L*. and *Rosa L*.) were found in all surveyed plots (Table S4).

## 4 Discussion

A systematic review by Huq and Deacon (2025) investigated the support for urban agriculture in terms of food provision, environmental protection, social cohesion, and citizens' health. The results of our research into the species of plants in gardens contribute to this topic and show how different functional plant groups (edible, medicinal, and ornamental) as well as melliferous plants promote the multifunctionality of the flora in allotment gardens and the delivery of ecosystem services by these gardens. The research also shows how the intensity of garden management (high vs. medium) influences the plant species richness of plant groups responsible for different ecosystem services.

A survey of plant species richness in allotment gardens revealed a predominance of ornamental and non‐native species in the plots. Most of the species identified were perennials. This is consistent with other field studies, such as those of front‐facing gardens in Chicago (USA) by Lowenstein and Minor (2016), which found that urban plant species tended to be perennials, ornamentals, and non‐natives. Recent studies in European allotments in Poland confirm the dominance of ornamental species and cultivated species richness, as well as a relatively high number of taxa cultivated in individual plots (Klepacki and Kujawska 2018).

Our study demonstrates that Berlin's allotment gardens contain a wide variety of plant species with diverse functions. Furthermore, the majority of garden plant species belong to more than one functional group. Native species make up only a small proportion. While the primary functions of garden areas are food production and recreation, a quarter of all garden species are weeds that are also used for decorative purposes. These results confirm the important role of allotment gardens as part of the multifunctional green infrastructure within urban ecosystems.

### 4.1 Multifunctionality and Ecosystem Service

The main strengths of the present study include a detailed survey of the garden flora, species identification and classification by type of use, including ornamentals, and the identification of native species. The study also aimed to explore the multifunctionality of garden plants, which could improve land use efficiency in urban allotment gardens. The results of this study could provide gardeners with environmental guidelines on which species to grow in their plots.

Most garden plants are grown for their ornamental value. The high proportion of ornamental plant species in garden plots makes garden areas attractive for visitors and serves an educational function for citizens. Cultivating plants used in traditional medicine provides an opportunity to preserve traditions, such as the preparation of herbal teas and infusions. Our results highlight the role of allotments in conserving native plant species and as a source of diverse plant species for food. We also suggest that most plant species grown in allotments are multifunctional: more than half of the total number of identified plant species are simultaneously melliferous and ornamental, melliferous and edible, or edible, medicinal and ornamental.

Spontaneous species also play an important role in garden biodiversity and can account for two‐thirds of all plant species, as demonstrated by a study in Leipzig, Germany (Cabral et al. 2017). In contrast, our study found that weeds accounted for just over 20% of the total number of species, while cultivated species accounted for more than 70%. However, our results are consistent with those of Cabral et al. (2017), which showed higher spontaneous (weed) species richness in comparison to high‐intensity managed plots.

Although most plant species in allotments are typically not native, studies show that allotments can be considered biodiversity hotspots for native species within urban green infrastructure (Borysiak et al. 2017). In our study, native species accounted for 9% of the total number of species, most of which were melliferous (pollen‐producing) plants.

The primary function of urban gardens is to grow fruit and vegetables. Consequently, gardens with a wide variety of fruit and vegetables can be an important local food resource (Sovová 2015). In line with the findings of Klepacki and Kujawska (2018) and Grafius et al. (2020), our results show that a wide variety of fruit and vegetables is cultivated in the plots, accounting for 18% of the total species count. Investigating yield variability from plot to plot would therefore be a worthwhile extension of this study, as poor cultivation practices can reduce yield, according to Woods et al. (2016). Some gardeners in the studied gardens use raised beds to increase yield and maintain soil fertility, including the use of their own organic compost, prepared either in classic piles or in specially designed containers, but these practices are not used in all plots. Foster et al. (2017) emphasize the importance of including floral resources in food‐growing gardens and allotments, and of providing a variety of flowering plants to cater for the different preferences of bumblebee species. In our study, the number of flowering plant species varied slightly between plots, ranging from 69% to 77%. There were plants from all useful groups: edibles, ornamentals, and weeds. Flowering hedges can also provide food for pollinators (Blanusa et al. 2019). In our study, hedges were represented by melliferous plants such as *Ligustrum vulgare* L. and *Pyracantha coccinea* in half of the surveyed plots.

Similar to the findings of Speak et al. (2015) and Clarke and Jenerette (2015), we observed high biodiversity in allotment gardens. This supports both food production and recreation for urban residents (cultural and provisioning ecosystem services) as well as biodiversity conservation (regulatory and maintenance ecosystem services). By surveying and analyzing plant species richness at the genus and species level, we build on existing allotment garden studies (Cabral et al. 2017; Borysiak et al. 2017), which are based on this approach.

### 4.2 Habitat for Pollinators

Previous studies have examined the impact of floral diversity on ecosystem services (ES) and the nativeness of garden flora. However, they have not focused strictly on melliferous or polliniferous plants, nor have they compared the plant diversity of intensively and non‐intensively managed plots. In the present study, each plant species was categorized as melliferous or non‐melliferous, including ornamentals, that is, plants that are pollinated by insects and provide them with nectar and/or pollen. Ornamental plants can provide long‐term supplementary foraging resources for generalist pollinator communities, which are characteristic of urban and suburban environments (Erickson et al. 2020). The positive effects of adding flowers to gardens are largely independent of the plant's origin, whether it is native or non‐native (Wenzel et al. 2020).

According to Hülsmann et al. (2015), flower‐rich parks and gardens can provide a continuous food supply that attracts bumble bees, even in isolated areas of the city centre, provided they contain a specific composition of plant species (e.g., Fabaceae and Lamiaceae). Flowers with longer corollas attract less common bumble bee species, especially those from the Fabaceae family. Lamiaceae and Boraginaceae., Plantaginaceae and Ranunculaceae families (Sikora et al. 2020). Our research showed that melliferous representatives of the Lamiaceae, Ranunculaceae and Fabaceae families accounted for 44 species, or 12% of the total number of species. The predominance of melliferous plant species among garden plants generally creates favorable conditions for insects that feed on nectar and pollen. However, only a small proportion of native species were found among honey plant species. According to some studies (Seitz et al. 2020), these native species may be more important for the conservation of native insect species.

The biodiversity of allotment gardens can be increased by planting a variety of flowering plants, with a preference for native and near‐native species. However, a selection of exotic plants should also be included to extend the flowering season and potentially provide resources for specialist species (Salisbury et al. 2015).

### 4.3 Medium Intensity vs. High Intensity Management

We hypothesized that plant species richness would vary between plots with different management intensities. Our results show that the intensity of allotment garden management shapes plant communities: the plant species richness of highly managed plots is lower than that of medium‐managed garden plots. Significant differences were found in the number of species from different plant groups. The greatest differences were observed in the number of native species, as well as in the number of plant species associated with regulatory and maintenance ecosystem services. Weedy plants were more prevalent in gardens with a medium management intensity. This finding is consistent with the work of Borysiak et al. (2017), who emphasized that gardeners' decisions are a key driver of plant diversity composition. With regard to invasive species, plots with medium management intensity showed a slightly higher richness of species belonging to this group than highly managed plots. All identified invasive species were ornamental plants, which is consistent with the findings of Potgieter et al. (2017), who reported that cultural ecosystem services, particularly aesthetics, are among the most frequently documented ecosystem services provided by alien plant species in urban areas of developed countries.

### 4.4 Global Impact

Allotment gardens should remain an important part of the urban ecosystem and be integrated into urban planning as multifunctional green spaces that support biodiversity. These spaces should provide food and cultural benefits, such as recreation and the preservation of folk traditions, while also providing shelter for pollinators, as confirmed by surveys of gardeners in Berlin (Dagmar and Dara 2023). This highlights the key role of allotment gardens in enhancing urban environmental resilience and supporting the targets of the EU Biodiversity Strategy 2030, such as reversing pollinator declines.

### 4.5 Limitation and Study Perspectives

A limitation of our study is that it focused only on plant richness, without considering the associated fauna or soil biota, and the number of plots was small. Additionally, we considered a limited number of ecosystem services, excluding types such as climate regulation and soil fertility maintenance. Future research should examine the interactions between organisms of different functional groups, such as the role of garden plants in preserving indigenous fauna, and should include a greater number of garden plots from all allotment associations in Berlin, in order to capture the full range of allotment garden ecosystem services and the differences between gardens. The present study is retrospective and examines the biodiversity of gardens in 2019. It would be valuable to survey the same gardens again in a few years' time to show the dynamics of overall biodiversity in garden plant communities and changes in species abundance within functional groups.

The small sample size prevented us from demonstrating the full statistical effect of allotment garden management intensity on plant biodiversity. Furthermore, the time and human resources available for the study did not allow us to calculate the area occupied by the most common species in order to quantify the benefits of ecosystem services. These gaps will be addressed in future research. Despite the above limitations, the field data provide unique insights into the plant species richness of Berlin's allotment gardens and could inform further studies on urban garden plant diversity.

Urban allotment gardens can play an important role in addressing several urban policy challenges, such as promoting stewardship of urban ecosystems, providing opportunities for recreation and healthy lifestyles, and fostering social cohesion (Camps‐Calvet et al. 2016).

Park et al. (2026) emphasize that urban garden management requires a flexible balance between biodiversity conservation, practical garden functions, and community interests. Recent evidence shows that vegetable gardening practices may positively contribute to urban biodiversity and pollinator‐supportive vegetation rather than conflict with biodiversity conservation goals (Collard et al. 2026). Gardens with high plant diversity can support cultural services, such as fostering local identity and nature experiences, as well as provisioning services, particularly food‐related ones, in urban areas (Fischer et al. 2019). Despite or because of their location, the cultivation of plants adapted to urban conditions can have a positive impact on urban biodiversity (Adams et al. 2020). Plant richness in gardens significantly contributes to the provision of ecosystem services. In this study, we sampled 377 species, including 273 melliferous species and 17 native species. This supports the suggestion by Wilson and Jamieson (2019) that increasing the abundance and richness of floral resources can partially compensate for the negative effects of urbanization on bees. We also conclude that allotments in Berlin play an important role in food production and in increasing the variety of vegetables consumed, with a total of 69 species, as well as an additional eight species that are both edible and ornamental. Allotment gardens are comparatively small spaces where gardeners and visitors can enjoy a variety of local and exotic flora.

In particular, urban children can learn about the plant and animal species living in their immediate environment (Ferrari et al. 2023). Therefore, allotments with a high diversity of ornamental plants can improve a neighborhood's recreational functionality, and vice versa, supporting the role of allotments as public recreational spaces. However, we have identified differences in plot management practices affecting plant diversity that require further investigation. We propose increasing the multifunctionality of allotment gardens by increasing phytodiversity and making gardening practices more affordable and environmentally friendly. Suggestions for improvement include reducing the frequency of lawn mowing and hedge trimming and including flowering honey plants in these areas. It is recommended that plant species that are beneficial to insects and have two or more functions are chosen. Furthermore, it has been demonstrated that less intensive management strategies can be employed to create high‐quality areas for flower‐visiting insects in urban green spaces (in line with Aguilera et al. 2019). The species composition of grasslands that were mown less frequently shifted from common mow‐tolerant turf species to typical meadow species. Our research has shown that, in plots with lower management intensity, a greater variety of native plant species are present, including *Lysimachia vulgaris L., Prunella vulgaris L., Malva sylvestris L., and Papaver rhoeas L., which simultaneously can serve as ornamental plants*. These findings support previous studies by Sehrt et al. (2020), who reported that reduced mowing is a simple and effective way to increase biodiversity in urban grasslands. Similarly, Chollet et al. (2018) found that lower mowing frequency increased plant diversity.

## 5 Conclusions

A total of 377 plant species were identified, including native species. Statistically significant differences between gardens with varying management intensities were found for groups of weed and native species, as well as groups of plants responsible for regulating ecosystem services. More than half of the identified plants were multifunctional, that is, ornamental for aesthetic and mental well‐being, useful for food and medicinal purposes, and melliferous, providing nectar and pollen for bees and other insects. We conclude that urban allotments can support pollinator populations due to the high richness of melliferous plant species. However, this capacity is strongly influenced by local plot management by the gardener. The number of melliferous plants varied particularly between gardens in the group of gardens with a high degree of management.

In this context, and to increase the quantity and quality of ecosystem services such as habitat provision, it is recommended that open spaces within and between gardens be sown with perennial melliferous native plants, with the cutting of flowering hedges postponed until early summer. Spots within plots can be created with plant mixes containing native flowering species that attract pollinators, such as *Lysimachia vulgaris L., Prunella vulgaris L., Malva sylvestris L., and Papaver rhoeas L*., which can also be used as ornamental plants. To create a more attractive habitat for wild bees or butterflies, gardeners need the advice of biologists to choose the best multifunctional plants. The potential spread of invasive species requires further monitoring, particularly for species included in the management lists of the German Federal Agency for Nature Conservation (BfN).

## Funding

The authors have nothing to report.

## Conflicts of Interest

The authors declare no conflicts of interest.

## Supporting information


**Table S1:** Descriptive for high and medium intensity management plot characteristics.

**Table S2:** Plant species per AG (allotment plot).

**Table S3:** List of identified plant species.

**Table S4:** Common species.

**Table S5:** IndVal results (all plant species across high and medium management plots).

**Table S6:** Occurrence of species in all gardens by family.

## Data Availability

The data that supports the findings of this study are available in the Supporting Information of this article.
