# Supplementary material for: Beautiful and Useful: Species Richness and the Ecosystem Services of Allotment Gardens in Berlin, Germany
Source: Plant Environ Interact. 2026 Jun 19;7(3):e70173. doi: 10.1002/pei3.70173 (PMC13282468; doi:10.1002/pei3.70173)
Supplement: Supplementary file 1 — Table S1: Descriptive for high and medium intensity management plot characteristics. [file PEI3-7-e70173-s005.docx]

*Table S1. Descriptive for high and medium intensity management plots characteristics*

|  | | high | medium |
| --- | --- | --- | --- |
| Age of garden association. years | Mean | 96.00 | 95.00 |
|  | Median | 78.00 | 87.00 |
|  | Std. Deviation | 24.90 | 23.58 |
|  | Minimum | 78.00 | 78.00 |
|  | Maximum | 128.00 | 128.00 |
|  | Interquartile Range | 45.00 | 42.00 |
| Total plot area. sq m | Mean | 381.40 | 406.50 |
|  | Median | 400.00 | 400.00 |
|  | Std. Deviation | 59.79 | 15.78 |
|  | Minimum | 277.00 | 396.00 |
|  | Maximum | 430.00 | 430.00 |
|  | Interquartile Range | 76.50 | 25.50 |
| Sealed area of the plot. % | Mean | 8.80 | 7.75 |
|  | Median | 7.00 | 6.50 |
|  | Std. Deviation | 3.83 | 3.59 |
|  | Minimum | 6.00 | 5.00 |
|  | Maximum | 15.00 | 13.00 |
|  | Interquartile Range | 6.50 | 6.25 |
